# Utility of AMACR immunohistochemical staining in differentiating Arias-Stella reaction from clear cell carcinoma of ovary and endometrium

**DOI:** 10.1186/s12885-023-10753-1

**Published:** 2023-04-11

**Authors:** Fatemeh Nili, Masoumeh Sadri, Fereshteh Ameli

**Affiliations:** 1 Department of Pathology, Cancer Institute, Imam Khomeini Hospital Complex, Tehran University of Medical Science, Tehran, Iran; 2 Postgraduate Doctorate Degree in Pathology (Pathologist), Department of Pathology, Cancer Institute, Imam Khomeini Hospital Complex, Tehran University of Medical Science, Tehran, Iran; 3 Department of Pathology, Cancer Institute, Imam Khomeini Hospital Complex, Tehran University of Medical Sciences, Tehran, Iran

**Keywords:** Arias-Stella reaction, Clear cell carcinoma, Alpha methyacyl CoA racemase (AMACR)

## Abstract

**Background:**

The Arias-Stella reaction is a hormone-related atypical endometrial change characterized by cytomegaly, nuclear enlargement, and hyperchromasia of endometrial glands; typically associated with intrauterine or extrauterine pregnancies or with gestational trophoblastic disease. Although differentiating the Arias-Stella reaction (ASR) from clear cell carcinoma (CCC) of the endometrium is usually straightforward, but differentiating ASR might be difficult if it occurs outside the setting of pregnancy, in extra-uterine sites or in older patients. The aim of this study was to determine whether P504S/Alpha Methyacyl CoA racemase (AMACR) immunohistochemical (IHC) staining can be used to differentiate ASR from CCC.

**Methods:**

Fifty endometrial ASR and 57 CCC samples were assessed by IHC staining with antibody for AMACR. The immunoreactive score (IRS) was based on total intensity score (no staining to strong scored as 0–3) + percentage score (0-100% categorized as 0–3) ranged from 0 to 6. Positive expression was considered as a total IRS exceeding 2.

**Results:**

The mean age of the patients in the ASR was significantly lower than that of CCC (33.34 ± 6.36 and 57.81 ± 11.64 years old, respectively, p < 0.001). The overall AMACR staining score was significantly higher among CCC compared to ASR groups (p = 0.003). The positive and negative predictive values for AMACR expression in detecting CCC from ASR were 81.1% and 57%, respectively.

**Conclusion:**

IHC staining for AMACR can be helpful and a member of discriminatory IHC panel when clinical or histologic features cannot facilitate the differential diagnosis between ASR versus CCC.

## Background

Arias-Stella reaction (ASR) is a reactive phenomenon in the endometrium, which occurs due to exposure to high-dose estrogen or progesterone during pregnancy, gestational trophoblastic disease or secondary to hormone administration [1]. This phenomenon was first described by Javier Arias-Stella in 1945. He described this reaction as a pseudoneoplastic glandular response of the female genital tract to excess sex hormones. ASR has five well known variant patterns, including minimal atypia, early secretory, secretory or hypersecretory; and regenerative or proliferative, also known as nonsecretory, as well as monstrous cell pattern. ASR can present with varying degrees of cytomegaly along with cytoplasmic clearing, vacuolization, nuclear enlargement, hyperchromasia, and changes in intraglandular papillary, as well as hob-nailing [2].

Endometrial clear cell carcinoma (CCC) is an uncommon variant of endometrial carcinoma. CCC resembles clear cell carcinoma in ovary and cervix. Based on the World Health Organization (WHO) classification of gynecological neoplasms, CCC should be diagnosed mainly based on histomorphological criteria. The typical presentation of CCC include cuboidal, polygonal or hobnail cells that have a clear-to-eosinophilic cytoplasm, which have a tubulo-cystic, papillary, or solid architecture [3]. Morphological features of CCC may considerably overlap with those of ASR, which may make challenges in their distinction. ASR diagnosis is usually uncomplicated in young pregnant patients. In contrast ASR diagnosis can be difficult in postmenopausal patients, and in patients who receive exogenous progestins due to known endometrial hyperplasia [4]. Immunohistochemistry (IHC) can be helpful in such difficult cases. Currently, HNF1β, Napsin A and Alpha-methylacyl-CoA racemase (AMACR) are the main suggested immunohistochemical markers in differentiating endometrial and ovarian CCCs [3].

The expression of Napsin A and HNF-1β is high in ASR. These markers were not helpful in separating ASR from CCC [1]. AMACR or p504s, is an evolutionarily conserved enzyme, which is important in branched-chain fatty acids metabolism [5]. AMACR was first detected based on cDNA library subtraction combined with high throughput microarray screening performed on normal and cancerous prostate tissues [6]. Later, anti-AMACR antibody soon was found to be a sensitive and specific tumor marker in prostate cancers [7, 8]. The most common application of anti-AMACR is detecting prostatic adenocarcinoma in routine practice. However, AMACR expression has been reported in extraprostatic neoplasms [9–11] and benign prostatic processes [12]. AMACR is also overexpressed in ovarian CCC, which is higher compared to other types of epithelial tumors [9]. AMACR expression has not been fully evaluated in ASR, but few studies have reported that ASR was associated with negative or low AMACR expression. Therefore, the objective of this study was to investigate AMACR expression among ASR and CCC, and evaluate its potential, as an IHC marker, in distinguishing ASR from CCC.

## Materials and methods

### Study design

This cross-sectional study was approved by the Ethics Committee of the Tehran University of Medical Sciences (IR.TUMS.IKHC.REC.1400.385). The study was conducted in Imam Khomeini Hospital, Tehran, Iran from March 2015 to March 2021.

### Study population

The electronic records of the Imam Khomeini Hospital Pathology Departments were screened to identify eligible patients. Patients with pathological diagnosis of CCC, preferably from endometrium, and ASR were included in this study. The diagnosis was confirmed by two pathologists by evaluating all Hematoxylin and Eosin slides with corresponding pervious IHC studies. Then, representative slides were selected and IHC study was performed on related paraffin blocks.

The clinicopathological variables were obtained from corresponding histopathology reports either via the Laboratory Information System or surgical department records.

Each patient was given a unique code to ensure the anonymity of the patient data. Blocks with inadequate tissue for IHC and those with incomplete medical records were excluded from the study.

### IHC study

IHC staining was performed using monoclonal rabbit anti-human AMACR/p504s rabbit monoclonal antibody + anti-human p63 mouse monoclonal antibody prepared in 10mM PBS, pH 7.4, with 0.2% BSA and 0.09% sodium azide. Acinar adenocarcinoma of the prostate was considered as positive control. After deparaffinization and rehydration, the sections were subjected to heat antigen retrieval technique. Immunostaining was performed based on the manufacturer standard protocol (Master Diagnostica, Spain).

### IHC staining interpretation

Semiquantitative scoring was performed to evaluate IHC staining using a high magnification (400x) light microscope on the unidentified samples using a 4-tiered system. Two pathologists (F.A and M.S) who were blinded to the clinicopathologic parameters and outcome of the patients independently evaluated the samples.

Scoring was based on overall stain intensity and the percentage of stained lesional cells. The intensity score was based on the estimated staining intensity. Intensity score include 0 (no staining), 1 (weak), 2 (moderate), and 3 (strong). The percentage score was based on the estimated fraction of positive-stained lesional cells. Percentage score was categorized as 0 (none), 1 (1-5%), 2 (6-49%), and 3 (50-100%). The total intensity score + percentage score defined as immunoreactive score (IRS) ranged from 0 to 6. Positive expression was determined as a total IRS exceeding 2 [13]. In case of discordance in staining degree between the pathologists, the issue was resolved by consensus between two pathologists.

### Statistical analysis

Data analysis was conducted using the statistical package for social sciences (SPSS) software version 16. Normality of continuous data was evaluated using the Kolmogorov-Smirnov test. Descriptive statistics were reported using mean and standard deviation (SD) for normally distributed or median and interquartile range (IQR) for non-normally distributed variables. Frequency and percentage were used to report categorical variables. The Fisher’s exact or Monte Carlo tests were used to compare the distribution pattern of categorical variables between diagnosis categories. The independent t-test or Mann-Whitney tests were used to compare mean or median value of continuous variables between diagnosis categories based on the normality of data. Binary logistic regression was used to evaluate the relationship between study variables and diagnosis with diagnosis categories as dependent variable and other study variables as independent variables. The receiver operating characteristics (ROC) curve analysis was used to evaluate the ability of AMACR in differentiating CCC from ASR. The level of statistical significance was considered as p < 0.05.

## Results

A total of 107 samples (57 CCC and 50 ASR samples) were evaluated. All ASR samples were endometrial curettage, but CCC group included 28 endometrial CCC (ECCC: biopsy and 19 hysterectomy specimens) and 29 ovarian CCC (OCCC: all oophorectomy specimens).

The mean age of the patients was 46.37 ± 15.52 years old. The mean age of the patients in the ASR and CCC groups were 33.34 ± 6.36 and 57.81 ± 11.64 years old, respectively. There was a significant difference in age between CCC and ASR groups (p < 0.001). The mean age of patients with OCCC (49.9 years) was significantly lower than ECCC (61.7 years) (P-value = 0.00).

The prevalence of endometrial and ovarian CCC was 49.12% and 50.88%, respectively.

**Fig. 1:**
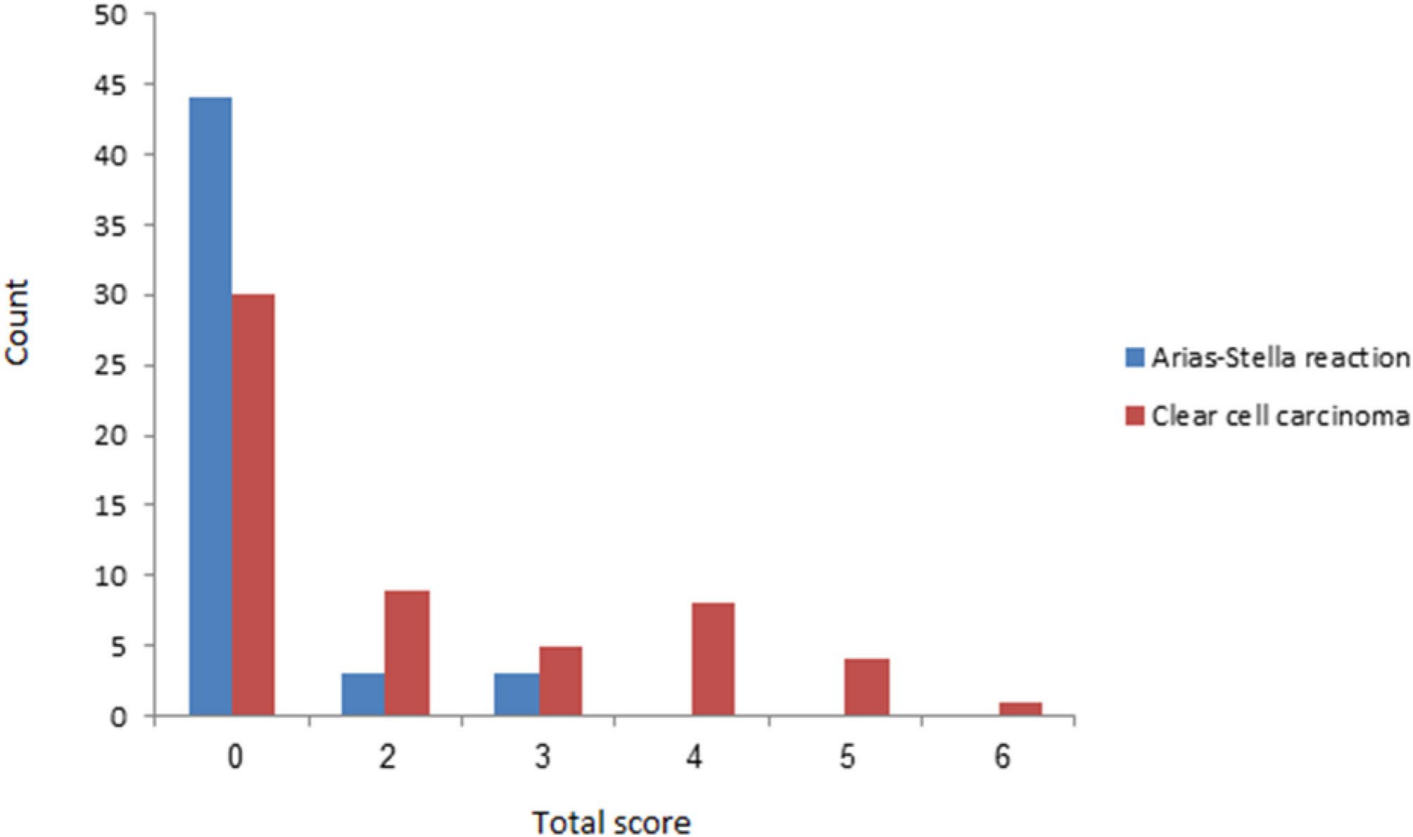
Comparison of the distribution pattern of total scores between Arias-Stella reaction and clear cell carcinoma groups

**Fig. 2:**
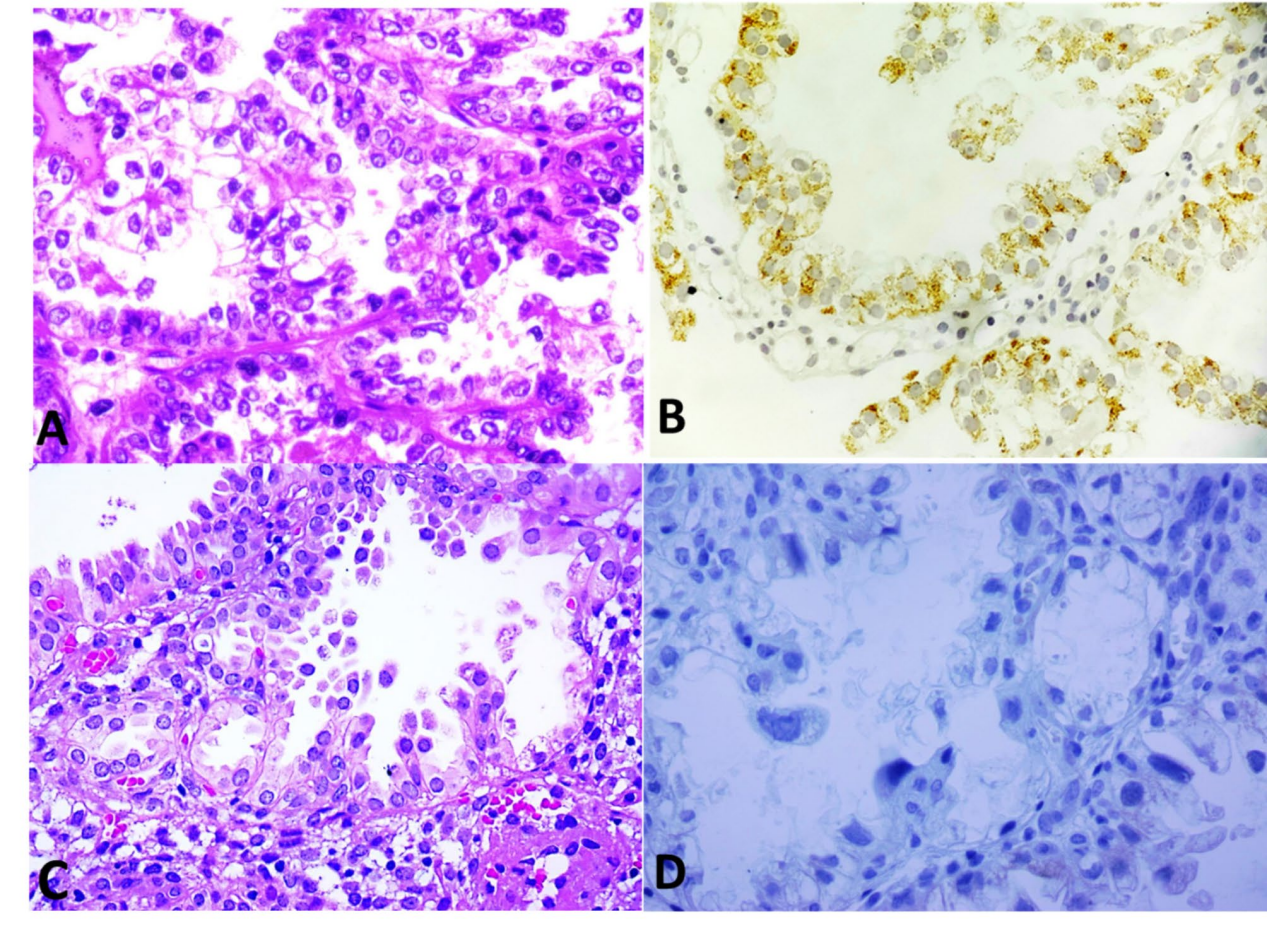
Comparison of AMACR expression between clear cell carcinoma and Arias-Stella reaction: (**A**) Clear cell carcinoma H&E x400, (**B**) IHC study AMACR on CCC x400 with strong positive expression (intensity score 3). (**C**) Arias-Stella reaction H&E x400, (**D**) IHC study AMACR on ASR x400 with negative expression (intensity score 0)

**Fig. 3:**
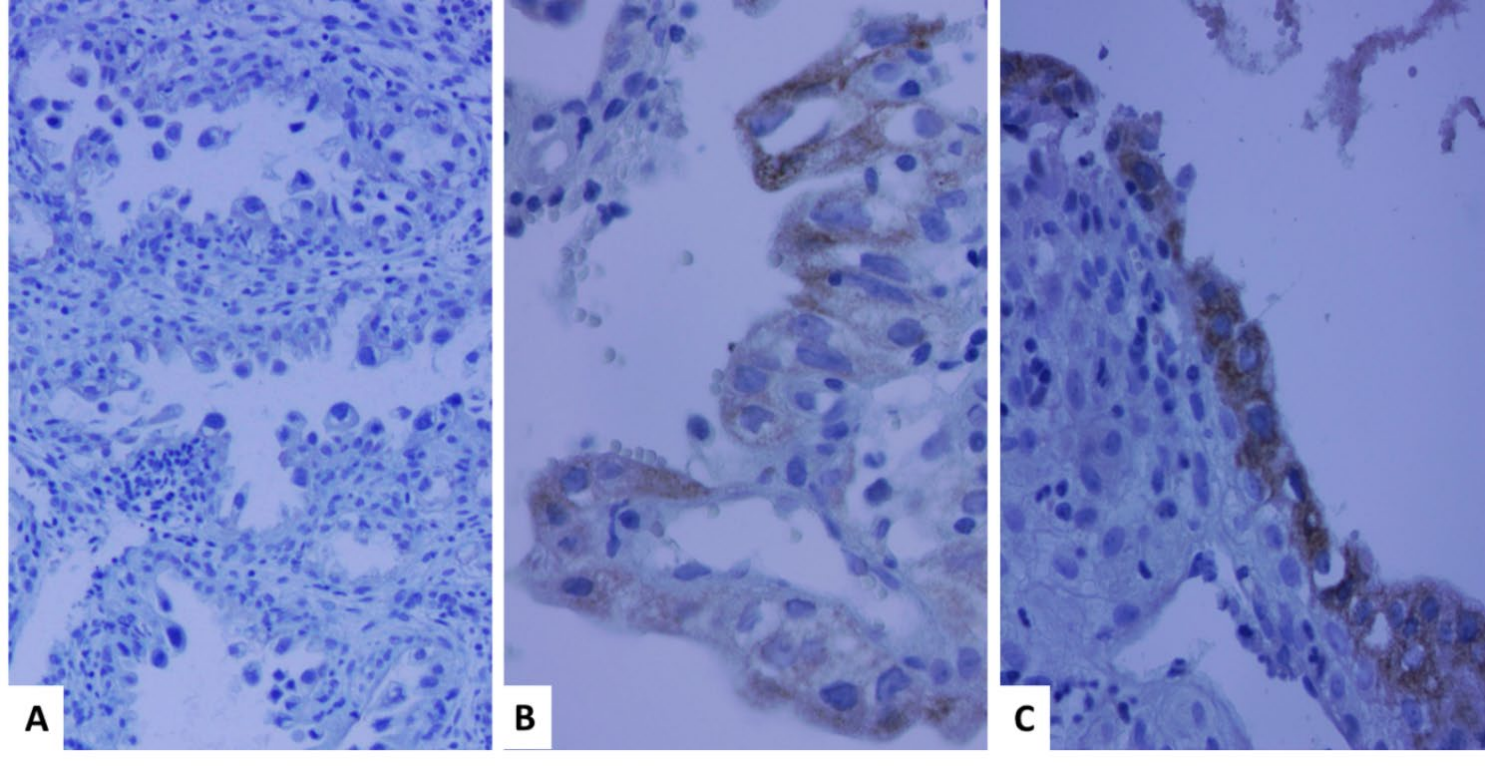
Comparison of AMACR expression with different intensity in Arias-Stella reaction (**A**) Intensity score 0 (no staining), (**B**) Intensity score 1(weak), (**C**) Intensity score 2 (moderate). Strong intensity was not detected

**Fig. 4:**
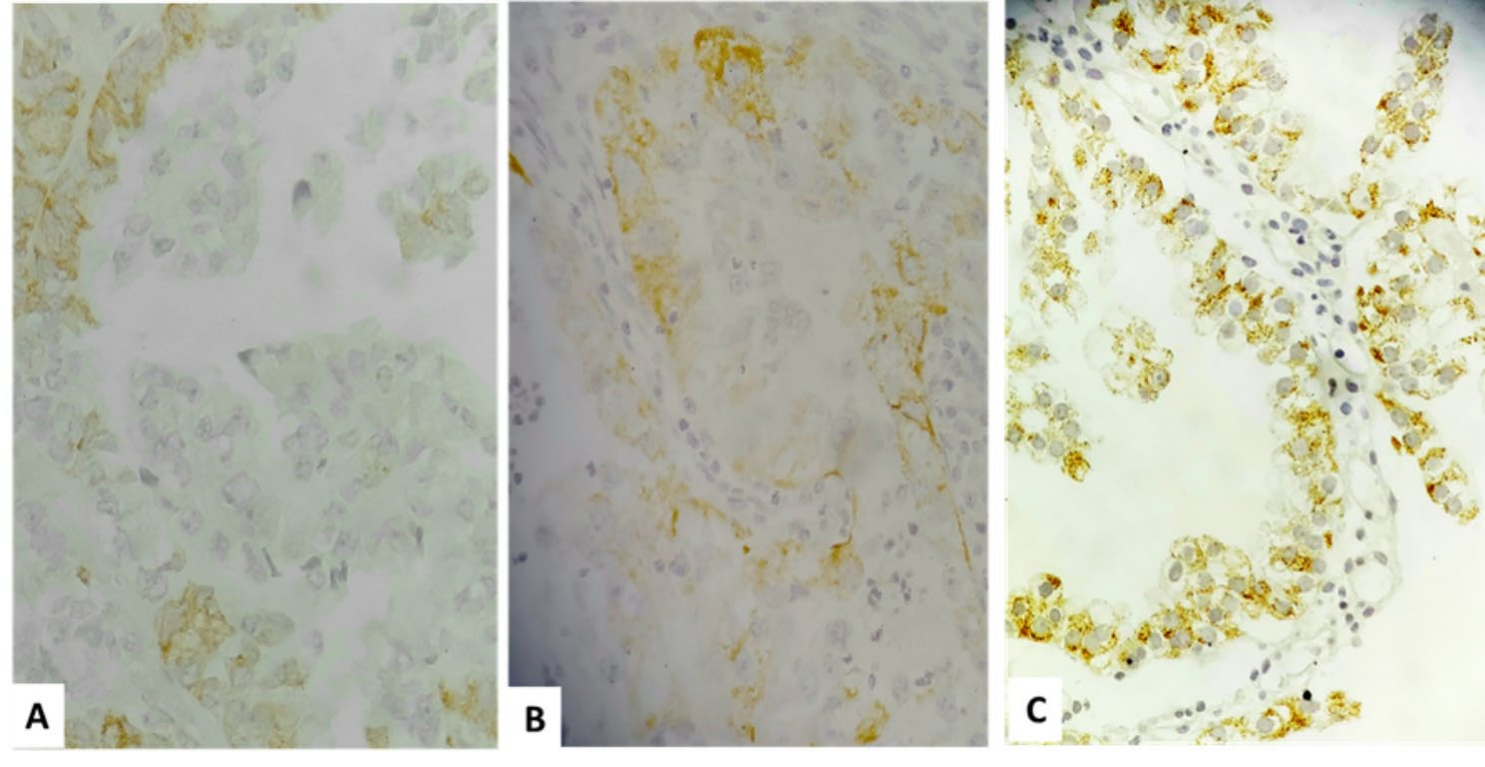
Comparison of AMACR expression with different intensity in Clear cell carcinoma (**A**) Intensity score 1 (weak), (**B**) Intensity score 2(moderate), (**C**) Intensity score 3 (strong)

Description of tumor characteristics of the samples in total population and their comparison between CCC and ASR groups are presented in Tables 1 and 2. The distribution patterns of percentage, intensity and total scores were significantly different between CCC and ASR groups (Table 1; Figs. 1, 2, 3 and 4). The mean total score in ASR and CCC groups were 0.30 and 1.59 respectively. There was a significant difference in IRS between the two groups (p = 0.003) indicating that IRS was significantly higher among CCC group compared to ASR group.

**Table 1:** Tumor characteristics of clear cell carcinoma

Tumor site	Frequency	Age (mean +/- SD)	Mean size	Stage (number of the cases)	
Ovary	29 (50.8%)	49.9+/-11.2	11.2+/-5.9	I	17
				II	2
				III	5
				IV	3
Endometrium	28 (49.1%)	61.7+/-13.1	4.9+/-2.9	I	7
				II	6
				III	3
				IV	6

**Table 2:** Tumor characteristics in total samples and their comparison between diagnosis groups

Variable		Total		Diagnosis				p
				Arias-Stella reaction		Clear cell carcinoma		
		Frequency	%	Frequency	%	Frequency	%	
Percentage score	0%	74	69.2%	44	88%	30	52.6%	< 0.001*†
	1–5%	16	14.9%	6	12.0%	10	17.2%	
	6–49%	13	12.1%	0	0.0%	13	22.8%	
	≥ 50%	4	3.8%	0	0.0%	4	7.4%	
Intensity score	Absent	74	69.2%	44	88%	30	52.6%	0.001*†
	Weak	21	19.6%	3	6.00%	13	22.8%	
	Moderate	15	14.0%	3	6.00%	12	21.0%	
	Strong	2	1.9%	0	0.0%	2	3.50%	
Total score	0	74	69.2%	44	88%	30	52.6%	0.003*†
	2	12	11.2%	3	6.00%	9	15.7%	
	3	8	8.4%	3	6.00%	5	8.7%	
	4	8	8.4%	0	0.0%	8	14.0%	
	5	4	3.7%	0	0.0%	4	7.01%	
	6	1	0.93%	0	0.0%	1	1.75%	

† The Monte Carlo test was used for the comparison

‡ The Fisher’s exact test was used for the comparison

* Significant difference

The results of binary logistic regression to identify the predictors of CCC are presented in Table 3. Among the study variables only age was significantly related to diagnosis (p = 0.013).

**Table 3:** Relationship between study variables and diagnosis

	p	OR	95% CI for OR	
			Lower	Upper
Percentage score	0.298	335.273	0.006	19093791.180
Intensity score	0.150	< 0.001	< 0.001	25.472
Age	0.019*	1.783	1.099	2.895

OR: Odds Ratio, CI: Confidence Interval

The binary logistic regression was used for the analysis

Total score was not included in the model due to redundancy

* Significant relationship

**Fig. 5:**
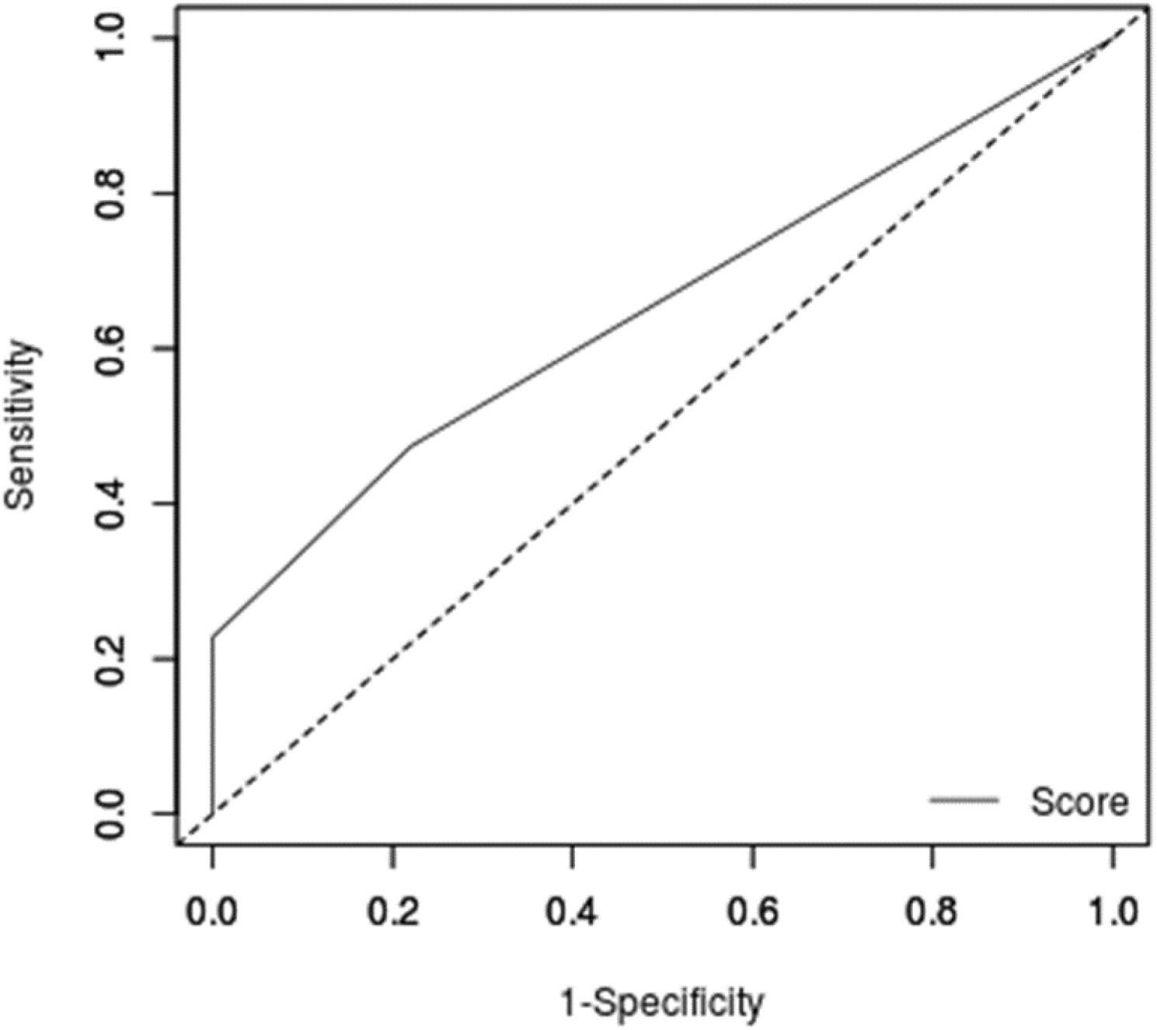
The ROC curve for Alpha-methylacyl-CoA racemase expression in diagnosing clear cell carcinoma

The ROC curve analysis was performed to evaluate the area under curve (AUC) for AMACR expression in distinguishing CCC from ASR cells. The AUC was 0.652 (95% CI for AUC: 0.565–0.738) indicating that AMACR expression could detect 65.2% of CCC cases. At the cut-off value of 2.0, AMACR expression could detect CCC with 47.4% sensitivity and 78.0% specificity. The positive and negative predictive values for AMACR expression in detecting CCC were 81.1% and 57%, respectively. The ROC curve is presented in Fig. 5.

## Discussion

In our study, we examined AMACR immunohistochemistry in a series of 50 ASR from endometrial site and 57 endometrial and ovarian CCC samples. Our results showed a significant difference in AMACR expression between CCC and ARS (p = 0.003) indicating that the IRS for AMACR expression was significantly higher among CCC compared to ASR groups.

In 2020, Ji et al. evaluated IHC markers to differentiate endometrial CCC from diagnoses that mimic its morphology, including ASR. The findings of their study added to the previous literature regarding the usefulness of Napsin A, HNF-1β, and estrogen receptor (ER) in CCC diagnosis. They demonstrated that arginosuccinate synthase (ASS1) and ER were the only markers that could potentially discriminate CCC from ASR. They reported that Napsin A and HNF-1β were highly expressed in ASR, which was similar to CC, but ER had 100% sensitivity and 88.2% specificity and ASS1 had 63.6% sensitivity and 95.1% specificity for diagnosing ASR [1]. Pors et al. (2019) surveyed AMACR expression in a series of 55 endometrial/cervical CCC and reported that 75% of CCC cases were AMACR stained and the staining intensity was more likely to be strong and diffuse [14]. Fadare et al. also showed that the sensitivity and specificity of AMACR expression in classifying CCC were 75% (95% CI: 0.61–0.86) and 79% (95% CI: 0.66–0.88), respectively, with an odds ratio of 11.62 (95% CI: 5–28, p < 0.001), and an AUC of 0.79 (95% CI: 0.68 to 0.88). These findings indicated a strong association between AMACR expression and CCC that make AMACR as a relatively robust diagnostic test [15]. However, the practical utility of AMACR expression evaluation may be limited by the focal nature of its expression, as focal expression is seen n in 32% of AMACR-positive CCC cases, as well as its expression in 15–22% of the non-CCC histotypes. AMACR expression of AMACR was reported to be negative in ASR cases [15].

Russell Vang et al. (2004) suggested that IHC staining for Ki-67 and p53 may help distinguish endometrial ASR from CCC and other of high-grade carcinoma types [16]. To the best of the author’s knowledge, no study has been conducted to investigate AMACR expression in ASR cells and its comparison to CCC. Our study showed that the AMACR can be a potentially useful marker for distinguishing ASR from endometrial CCC. However, in view of low expression of AMACR in CCC cases in our study compared with previous studies, different clones of this antibody should be investigated to identify the best colon.

Among the study variables only age was significantly related to CCC diagnosis (p = 0.013). This finding indicated that with increase in age the risk of CCC diagnosis increased by 78.3%. This could be explained as most ASR cases occur at young age and are associated with pregnancy or hormone therapy.

The utility of AMACR as a single marker or as a putative complementary marker of a panel in distinguishing CCC from ASR needs to be compared with ER and ASS1 in further studies. The power of the regression analysis was 75%, which was smaller than the estimated power of the study (80%) and could be considered as a limitation of the study. Therefore, it is probable that the observed relationship might not remain significant in larger sample size. As this study included all the eligible samples in a single pathology laboratory, further multicenter studies are required to justify the findings of this study.

## Conclusion

In summary, this study described potential utility of AMACR as a diagnostic adjunct in distinguishing CCC from ASR. However, the utility of AMACR as a single marker or as a part of a panel with other immunohistochemical markers, including ER and ASS1, should be further evaluated.

## Data Availability

The datasets generated and analysed during the current study are not publicly available but are available from the corresponding author on reasonable request.
